# Design of a Multi-Beam Switching Antenna Loaded with a Square Metasurface

**DOI:** 10.3390/mi16111298

**Published:** 2025-11-20

**Authors:** Ningchuan Liu, Lin Huang, Lingxiao Huang

**Affiliations:** 1 School of Electronic Science and Engineering, University of Electronic Science and Technology of China, Chengdu 611731, China; lncjsw1@163.com; 2 Zhongyuan Electronic Group Co., Ltd., Wuhan 430070, China

**Keywords:** metasurface, multi-beam, low profile

## Abstract

Multi-beam and beam-scanning antennas enable extensive communication coverage while mitigating multipath fading and enhancing spectrum utilization efficiency. This paper presents a transmissive metasurface antenna design, which utilizes a microstrip square-ring patch antenna with four feed ports as the excitation source. A 7 × 7 square patch metasurface is positioned above the feed source, facilitating the generation of four independently steerable beams by switching activation among the four feed ports. Operating at 12.6 GHz, the antenna achieves a gain of 10.4 dB. The 3 dB beamwidth of the beams from all four ports exceeds 23°. The proposed design offers advantages of structural simplicity, low profile, and cost-effectiveness. By leveraging transmissive metasurfaces, this approach combines the benefits of low profile and low cost with flexible manipulation of electromagnetic wave radiation, thereby providing a novel methodology for designing multi-beam communication antennas.

## 1. Introduction

With the rapid deployment of 5G networks and the high-speed advancement of satellite communications, multi-beam and beam-scanning antenna systems characterized by low cost, low power consumption, high reliability, easy deployment, and easy integration have become crucial in building integrated network communications across multi-dimensional domains including space, sky, terrestrial, and maritime environments [1,2]. Multi-beam and beam-scanning antennas not only address issues in wireless channel environments such as path loss and multipath fading, but also enable deep integration between satellite communication networks and terrestrial 5G networks. This capability provides ubiquitous signal coverage unrestricted by terrain features, particularly in areas inaccessible to ground-based networks. Consequently, these antennas are widely employed in surveillance systems, UAV communications, satellite communications, and other fields.

The multi-beam radiation or beam-scanning capability of antennas is primarily achieved through phase modulation, amplitude modulation, or a combination of both. Various antenna types, including lens antennas, reflector antennas, phased array antennas, and metasurface-based beamforming antennas, can realize multi-beam radiation or beam scanning through different control methods [3,4,5,6,7].

Lens antennas feature continuous radiating apertures and primarily rely on geometric optics principles to achieve multi-beam radiation and wide-angle beam scanning. However, they suffer from drawbacks such as large volume, high profile, and significant dielectric loss. Current implementations of lens antennas mainly include Rotman lenses and Luneburg lenses.

By placing multiple feed sources near the focal point of a reflector antenna, beams with different pointing directions can be generated according to the generalized Snell’s law. However, beam scanning in reflector antennas tends to cause pattern distortion, increased sidelobe levels, and widened beamwidth, making them unsuitable for wide-angle multi-beam scanning.

Traditional phased array antennas achieve beam scanning by controlling the phase and amplitude of antenna elements through complex T/R (transmit/receive) circuit modules. These antennas are characterized by large volume, high manufacturing costs, and complex feeding networks, which hinder system miniaturization and lightweight design.

Metasurface-based beam control technology has gradually become a prominent research direction. Such metasurface antennas typically consist of one or multiple feed sources integrated with a metasurface, where the feed source is usually placed at one end or in the center of the metasurface. Since the metasurface and the antenna form an integrated structure, they are collectively referred to as metasurface antennas. Due to advantages such as low fabrication cost, low profile, and simple architecture, metasurface-based multi-beam and beam-scanning antennas have been widely applied in manipulating terahertz waves [8], microwaves [9], and visible light [10].

In recent years, the proposal of metasurfaces has attracted significant attention and research interest in both the physics and engineering communities [11,12,13,14]. Metasurfaces, composed of two-dimensional arrays of subwavelength artificial structures, enable flexible manipulation of the amplitude and phase of electromagnetic waves, thereby facilitating antenna multi-beam radiation or beam-scanning capabilities [15,16,17]. Their emergence provides a novel approach for beam formation and control, contributing to the development of integrated and lightweight modern wireless communication systems.

Currently, most multi-beam scanning antennas studied domestically and internationally utilize horn antennas as feeds. The considerable distance between the feed antenna and the metasurface in these designs often results in antenna systems with high profiles [18,19]. This paper employs a combination of a microstrip feed antenna and a metasurface to simultaneously achieve the advantages of a low profile and low cost, while maintaining flexible control over electromagnetic radiation.

The transmissive metasurface antenna designed in this work uses a microstrip square-ring patch antenna with four feed ports as the excitation source. A 7 × 7 square patch metasurface is loaded above the feed source, enabling the generation of four beams steered to different directions by switching activation among the four feed ports. The antenna operates at 12.6 GHz with a achieved gain of 10.4 dB.

## 2. Microstrip Antenna and Metasurface Design

**Figure 1:**
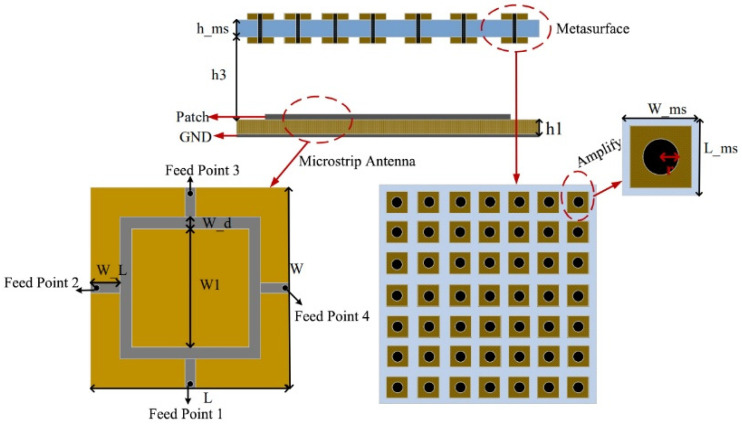
Overall configuration of the metasurface antenna.

The overall structure of the metasurface antenna is illustrated in Figure 1, comprising a microstrip feed antenna and a square patch metasurface, operating at 12.6 GHz. The microstrip ring radiating patch (silver portion) is etched on an FR4 dielectric substrate (yellow portion) with a loss tangent of 0.01 and a relative permittivity of 4.4. The substrate thickness is 1.5 mm, with a ground plane on its bottom layer. The overall dimensions of the feed antenna are 56 mm × 56 mm × 1.5 mm.

The square patch metasurface elements are etched on an FR4 substrate (blue portion) with a relative permittivity of 4.4. The brown segments represent the metallic patches of the metasurface, while the black parts indicate the metallic shorting pins connecting the upper and lower patches of the metasurface. The metasurface unit cell has a period of *W_ms* = 8 mm, approximately 0.33λ_0_ (where λ_0_ is the wavelength at the center frequency). The entire metasurface consists of a 7 × 7 array of square patches. The central integration of black shorting pins in the metasurface unit cells aims to suppress inter-element electric field coupling and enhance beam performance.

The metasurface is positioned directly above the feed antenna at a distance of h_3_ = 5 mm. The air layer between the feed antenna and the metasurface can be replaced by foam. Through the design and optimization of the metasurface antenna parameters using CST Studio Suite, the final dimensions of the multi-beam antenna based on the transmissive metasurface designed in this chapter are summarized in Table 1.

## 3. Simulation and Analysis of the Metasurface Antenna

### 3.1. Design and Simulation of the Metasurface Unit Cell

**Figure 2:**
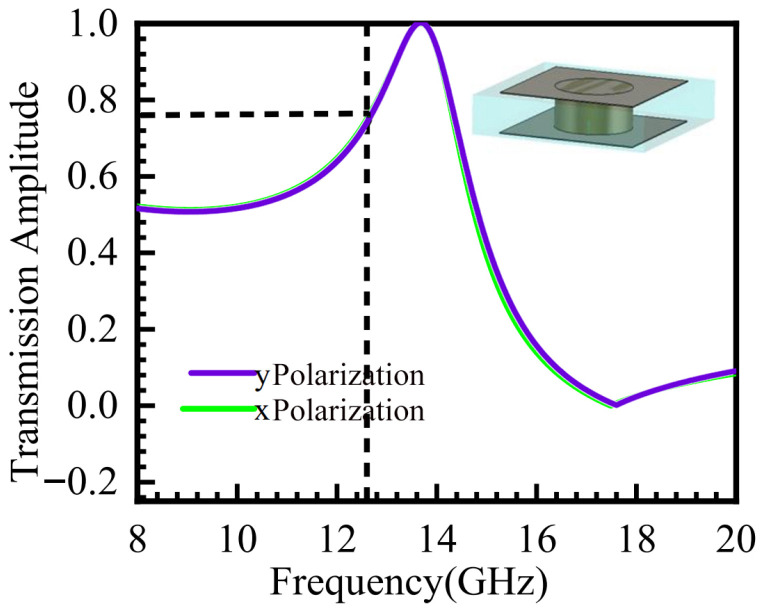
Transmission Characteristics of the Unit Cell under Electromagnetic Wave Incidence.

The structure of the metasurface unit cell is shown in Figure 1 above. The transmission amplitude of the unit cell was simulated using CST software. Due to the structural symmetry of the metasurface unit, the amplitude response is identical for x-polarized and y-polarized incident waves. The simulation results are presented in Figure 2.

**Figure 3:**
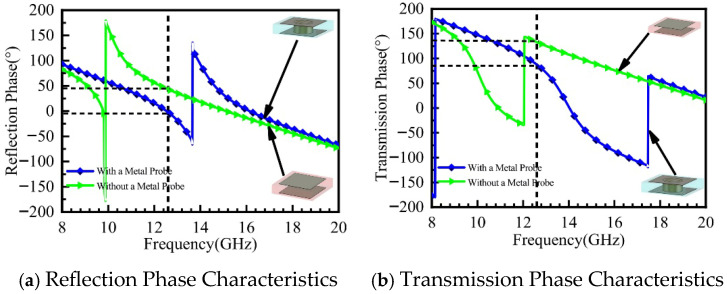
Phase Response of the Metasurface Unit Cell.

Figure 3a,b present the simulated phase results of the metasurface unit cell. The blue curve in Figure 3a shows the reflection phase characteristics of the unit cell with a metal pin, while the green curve represents those without a metal pin. Simulation results indicate that at the operating frequency of 12.6 GHz, the reflection phase of the unit cell with the metal pin is φr_0_ = −1.65°, and that without the metal pin is φr_1_ = 50°. The blue curve in Figure 3b illustrates the transmission phase characteristics of the unit cell with the metal pin, and the green curve corresponds to the unit cell without the metal pin. The results show that the transmission phase of the unit cell with the metal pin is φt_0_ = 89°, while that without the metal pin is φt_1_ = 133°.

**Figure 4:**
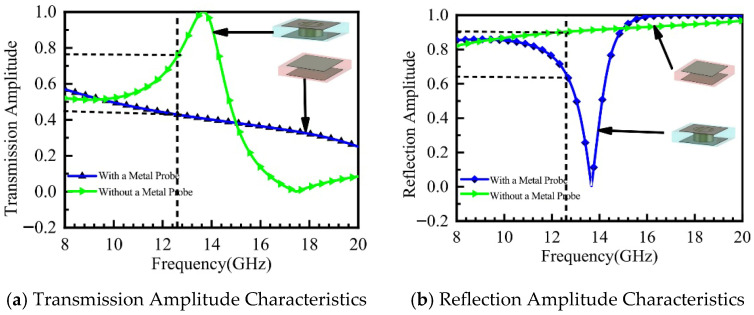
Amplitude Response of the Metasurface Unit Cell.

Figure 4a,b display the simulated amplitude results of the metasurface unit cell. The results indicate that the transmission coefficient of the unit cell without the metal pin is Pt_1_ = 0.4, whereas that of the unit cell with the metal pin is Pt_0_ = 0.75. As can be observed from Figure 4b, the reflection amplitude of the unit cell with the metal pin is Pr_0_ = 0.86, while that without the metal pin is Pr_1_ = 0.6.

Based on the analysis of the unit cell’s amplitude and phase characteristics, the integration of the metal shorting pin enables the transmission phase φt_0_ of the metasurface unit cell to approach 90°. The phase difference between the metasurface and the ground plane satisfies the π/2 condition, which facilitates in-phase superposition and radiation of electromagnetic waves. Compared to the unit cell without a metal pin, the unit cell with the metal pin exhibits a higher transmission amplitude while maintaining favorable reflection characteristics, making it advantageous for constructing resonant cavity antenna structures.

**Figure 5:**
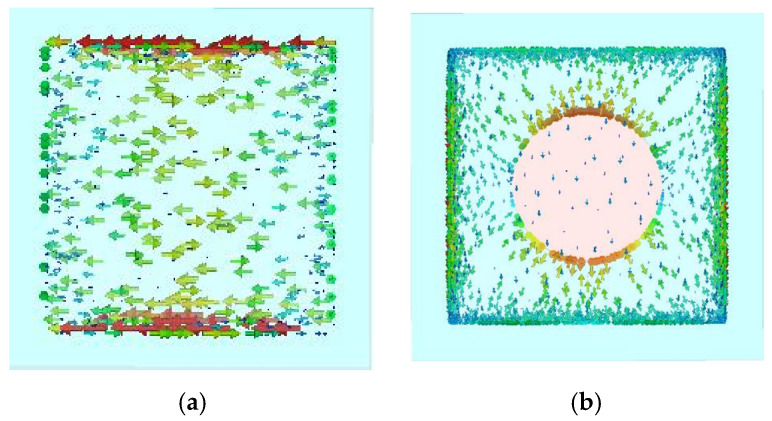
Current Distribution of the Metasurface Unit Cell. (**a**) Metasurface Unit Current Distribution without Metal Probe. (**b**) Metasurface Unit Current Distribution with Metal Probe.

Figure 5a,b show the current distribution on the metasurface unit cell at the operating frequency of 12.6 GHz. Figure 5a depicts the surface current distribution on the square patch unit without a metal pin. It can be observed that the current density is highest at the edges of the metasurface patch, which can easily lead to electric field coupling between adjacent unit cells. This coupling excites surface waves, causing beam scattering and increased sidelobe levels. Figure 5b illustrates the surface current distribution on the unit cell with a metal pin. Here, most of the current flows from the edges towards the center, resulting in high current density at the central pin and low current density at the edges, thereby suppressing surface waves. Furthermore, the alteration in the metasurface current distribution leads to corresponding changes in the amplitude and phase characteristics of the unit cell. Analysis of the amplitude-phase characteristic curves of the metasurface unit cell indicates that the unit with the metal pin exhibits a superior amplitude response.

**Figure 6:**
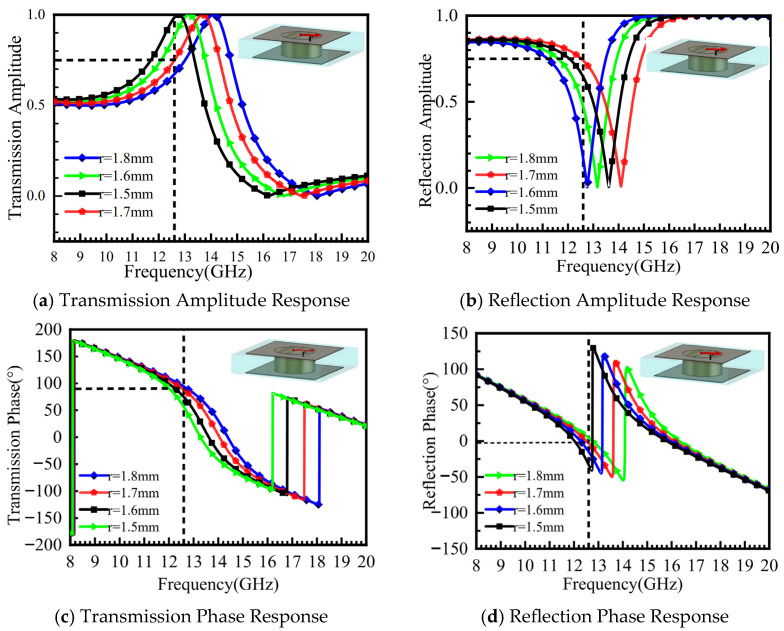
Effect of Metal Pin Radius r on Amplitude and Phase Characteristics of the Unit Cell.

Based on the surface current distribution of the metasurface unit cell in Figure 5b, it can be observed that the current is concentrated around the metal pin, flowing from the upper surface into the pin. Adjusting the radius of the pin alters the phase response of the metasurface. The amplitude and phase characteristics of the unit cell as a function of the metal pin radius (r = 1.5 mm, 1.6 mm, 1.7 mm, and 1.8 mm) are shown in Figure 6. As seen in Figure 6a, the maximum transmission amplitude Pt = 0.9 is achieved at r = 1.5 mm. Figure 6b shows that the reflection amplitude Pr reaches its minimum value of 0.2 at this radius, while Figure 6c indicates a reflection phase φr = −43° and a transmission phase φt = 50°.

Therefore, the condition for in-phase superposition of the reflected and transmitted waves cannot be satisfied. When r = 1.8 mm, Figure 6d shows that the transmission phase φt = 90°, and Figure 6b indicates that the reflection amplitude Pr reaches its maximum value of 0.75. However, the transmission amplitude Pt is minimized at 0.63. When the transmission amplitude is maximized and the transmission phase φt ≈ 90°, the phase difference between the reflecting surface and the ground plane is π/2. This enables in-phase superposition and radiation of the reflected and transmitted waves. After parameter optimization, the final radius is determined to be r = 1.7 mm. At this value, the transmission amplitude Pt = 0.75, the reflection amplitude Pr = 0.86, the transmission phase φt ≈ 90°, and the reflection phase φr = −23°, which is close to 0°.

### 3.2. Design and Simulation of the Metasurface Antenna

**Figure 7:**
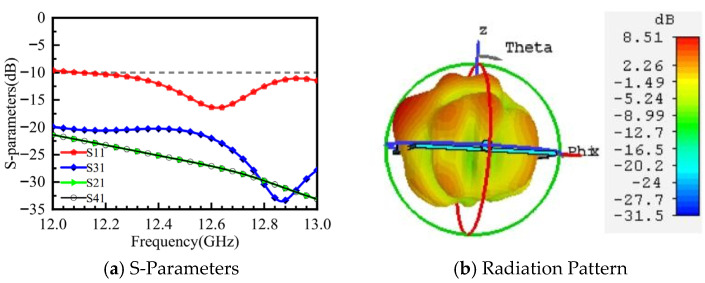
Simulation Results of the Microstrip Antenna.

Taking port 1 excitation as an example, the microstrip feed antenna without the loaded metasurface was simulated. The simulation results for S11, isolation, and the 3D radiation pattern are shown in Figure 7. In Figure 7a, the blue region indicates an impedance bandwidth of approximately 12.1–13 GHz. At the operating frequency of 12.6 GHz, the isolation between port 1 and the other three ports is better than −20 dB. Figure 7b shows that the microstrip antenna has a gain of 8.5 dB. The main beam is directed away from the z-axis, but the beam is scattered, with energy not concentrated and relatively high sidelobe levels. Therefore, a metasurface is placed above the feed antenna to form a resonant cavity structure with the antenna, enabling the antenna beam to radiate in a specific direction with low sidelobes and high gain in the main lobe.

**Figure 8:**
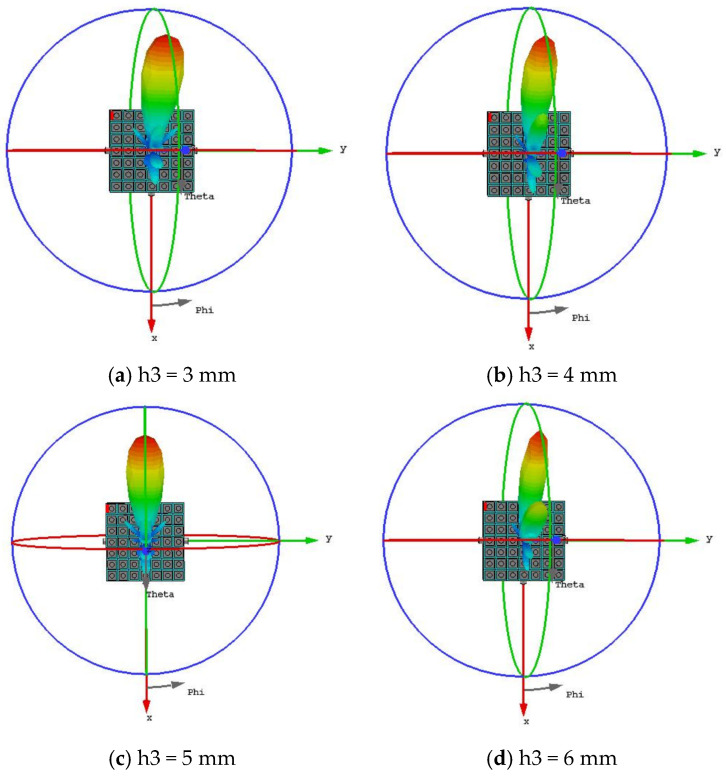
Radiation Patterns of the Metasurface Antenna under Different Cavity Heights.

The microstrip antenna loaded with the metasurface was simulated. Since the cavity height h_3_ significantly affects the beam performance, Figure 8a–d present the 3D radiation patterns for different values of h_3_ to provide a more intuitive understanding of its impact. When h_3_ = 3 mm, the gain is 9.37 dB, the half-power beamwidth (HPBW) is 23.7°, and the beam direction is (φ, θ) = (0°, −52°). However, it exhibits a high sidelobe level, numerous minor lobes, and low gain. As h_3_ = 4 mm, the gain is 10 dB, the HPBW is 22.3°, and the beam direction is (φ, θ) = (0°, −52°). Multiple sidelobes are observed, along with a trend towards multi-beam formation. While h_3_ = 6 mm, the gain is 10 dB, the HPBW is 22°, and the beam direction is (φ, θ) = (0°, −51°). The number of minor lobes increases with a high sidelobe level, and the multi-beam trend becomes more pronounced. When h_3_ = 5 mm, the antenna achieves the highest gain of 10.4 dB with a low sidelobe level. The beam steering angle is (φ, θ) = (0°, −52°) with a 3 dB beamwidth of 23.7°. Based on comprehensive analysis, the cavity height was ultimately determined to be h_3_ = 5 mm, as this configuration yields the highest antenna gain and the lowest sidelobe level.

**Figure 9:**
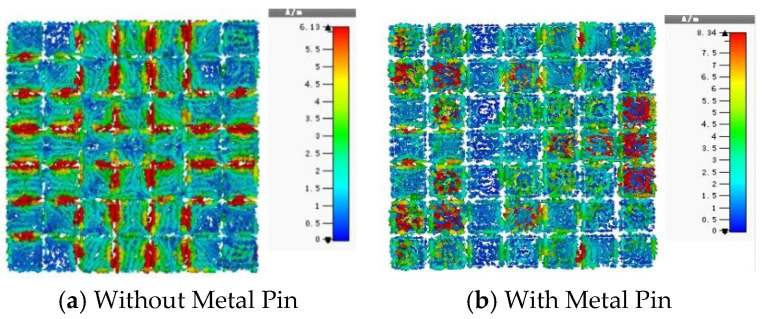
Current Distribution on the Metasurface (12.6 GHz).

**Figure 10:**
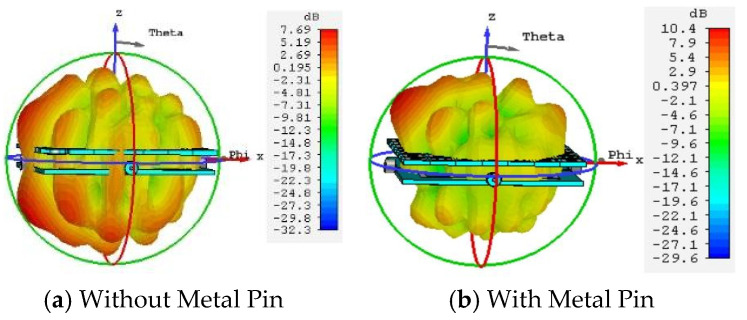
3D Radiation Pattern of the Metasurface Antenna (12.6 GHz).

The previous section provided a detailed analysis of the amplitude and phase characteristics of the metasurface unit cell, both with and without the metal pin. To further validate the impact of the pin on the antenna’s beam performance, a co-simulation of the metasurface and the microstrip feed source was conducted. Figure 9 shows the current distribution on the metasurface obtained from this co-simulation. Figure 10 presents the 3D radiation patterns of the metasurface antenna with and without the metal pin. In Figure 9a, the surface current is concentrated at the edges of each metasurface unit. The electric field at the center of the unit is the weakest, leading to electromagnetic coupling between adjacent units. This readily excites surface waves, resulting in significant scattering of the electromagnetic waves and an inability to focus the beam. As can be intuitively seen from Figure 10a, the metasurface antenna without the metal pin exhibits low gain, significant beam scattering, and numerous sidelobes. In contrast, the current distribution and 3D radiation pattern for the metasurface with the metal pin are shown in Figure 9b and Figure 10b, respectively. Due to the introduction of the metal pin, the current flows from the edges of the unit into the pin and then out from the center of the lower surface patch. Consequently, the current density at the edges of the metasurface unit is reduced, the electric field strength is weaker, and the beam can be more easily focused.

**Figure 11:**
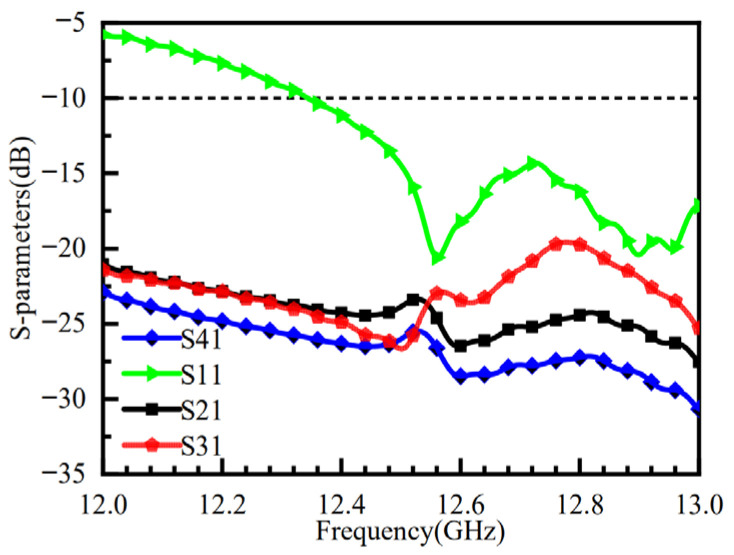
S-Parameters of the Metasurface Antenna.

Figure 11 shows the simulated S11 and port isolation results after loading the metasurface. The impedance bandwidth is improved with the metasurface, showing a −10 dB impedance bandwidth ranging from 12.5 to 13 GHz, and the isolation between all ports is better than −20 dB. (The S-parameter results for the other three ports are consistent with those of port 1 and are not analyzed in detail here.)

**Figure 12:**
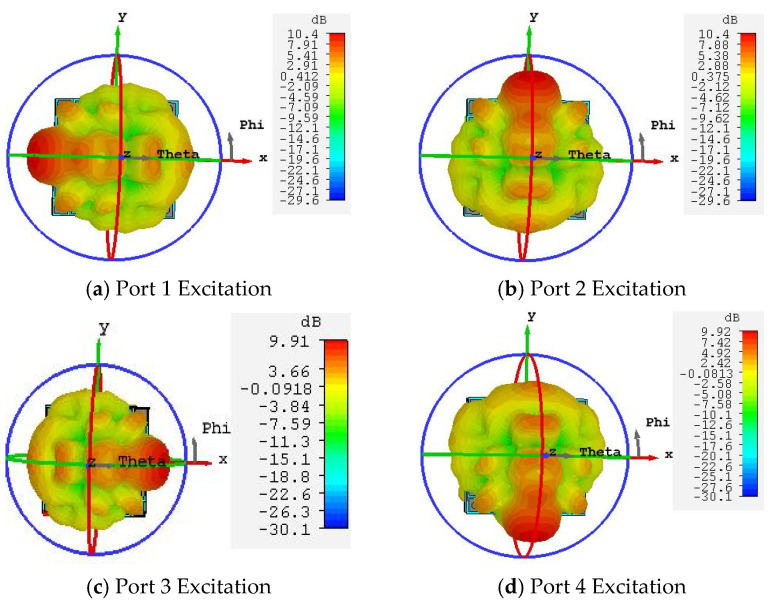
3D Radiation Patterns of the Metasurface Antenna with Different Port Excitations.

**Figure 13:**
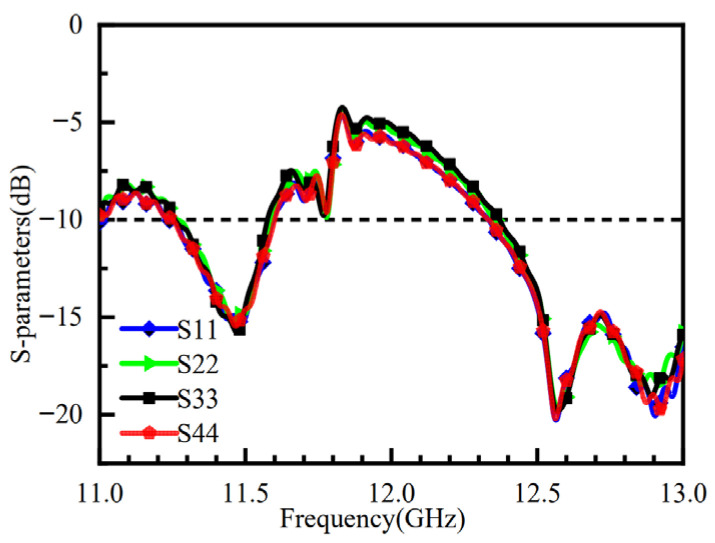
S-Parameters of the Metasurface Antenna with Different Port Excitations.

The electric field distribution induced on the metasurface varies when different feed ports are excited, leading to corresponding changes in the phase distribution across the metasurface. When the electromagnetic waves radiated from the feed source illuminate the metasurface, the antenna can generate four beams steered in distinct directions. Figure 12 presents the 3D radiation patterns for different port excitations: Figure 12a–d correspond to the excitation of port one, port two, port three, and port four, respectively. The results demonstrate that, when port one is excited, the beam points to (φ, θ) = (180°, 52°) with a 3 dB beamwidth of 23.9° and a gain of 10.4 dB. When port two is excited, the beam points to (φ, θ) = (90°, 51°) with a 3 dB beamwidth of 23.9° and a gain of 10.4 dB. When port three is excited, the beam points to (φ, θ) = (0°, 52°) with a 3 dB beamwidth of 23.7° and a gain of 9.91 dB. When port four is excited, the beam points to (φ, θ) = (270°, 52°) with a 3 dB beamwidth of 23.7° and a gain of 10.4 dB. The antenna’s four beams exhibit substantial steering angles, enabling coverage over a wide area. Furthermore, the impedance bandwidth remains consistent when sequentially exciting each of the four ports. The simulated reflection coefficients for each port are shown in Figure 13.

## 4. Fabrication and Measurement of the Metasurface Antenna

**Figure 14:**
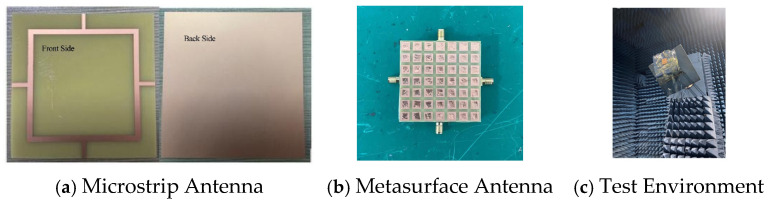
Fabricated Metasurface Antenna and Test Environment.

To validate the accuracy of the simulation results, the designed metasurface antenna was fabricated, and its S-parameters and gain were measured. The physical antenna and the test setup are shown in Figure 14. The air layer between the microstrip antenna and the metasurface was replaced with foam. The metasurface antenna was mounted on a turntable in an anechoic chamber, and the radiation pattern was measured using an NSI-2000 measurement system. The S11 and port isolation of the fabricated antenna were measured using a vector network analyzer.

**Figure 15:**
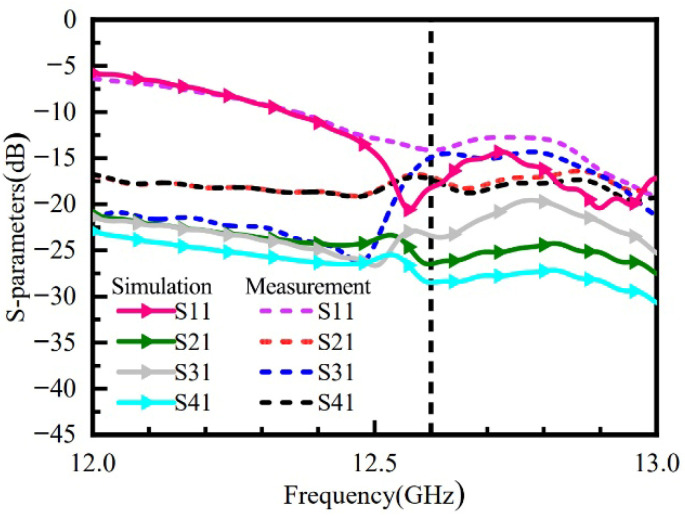
Measured S-Parameter Curves of the Metasurface Antenna.

Figure 15 shows a comparison between the simulated and measured results for S11 and port isolation. The measured results indicate that at the operating frequency of 12.6 GHz, the resonance depth is −15 dB, and the measured isolation between all ports is better than −15 dB. Some discrepancies exist between the measured and simulated S-parameter curves. These can be attributed to several factors: fabrication tolerances in the antenna and metasurface, environmental influences during testing, as well as potential inaccuracies due to equipment aging or calibration errors.

**Figure 16:**
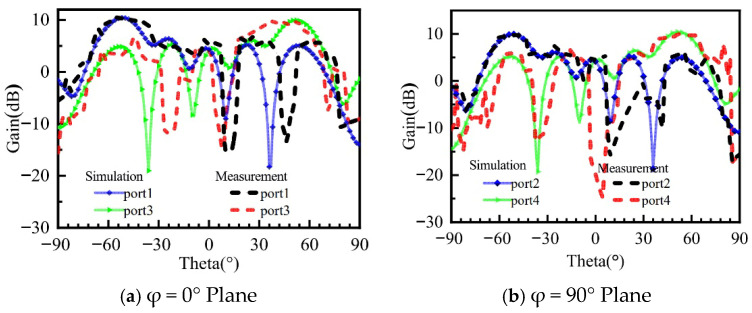
Comparison of Simulated and Measured Gain for the Metasurface Antenna.

Figure 16 presents a comparison between the simulated and measured 2D radiation patterns obtained by sequentially exciting the four feed ports. In Figure 16a, it can be observed that when port one is excited, the measured beam steering angle is (φ, θ) = (0°, −50°), with a maximum gain decrease of 1 dB and a sidelobe level increase of 1.5 dB compared to simulations. When port three is excited, the measured beam steering angle is (φ, θ) = (0°, −47°), with a gain decrease of 0.7 dB. In Figure 16b, when port two is excited, the measured beam steering angle matches the simulation at (φ, θ) = (90°, −52°). However, the sidelobe level increases by 1 dB. When port four is excited, the measured beam steering angle is (φ, θ) = (90°, 50°), accompanied by a significant increase in the sidelobe level and a gain decrease of 1.5 dB. The primary reason for the gain degradation is the need to solder SMA RF connectors to each port during physical testing. Limitations in my soldering technique and the use of non-customized SMA connectors have resulted in some discrepancies between the physical prototype and theoretical simulations. For future work, researchers may consider procuring customized high-frequency SMA connectors and employing professional soldering services, budget permitting, to minimize the deviation between measured and simulated results. Analysis of the simulated and measured results indicates that during the physical testing process, the measured gain values are slightly lower than the simulated values, and there are some inaccuracies in the beam steering angles. These discrepancies are likely due to manufacturing tolerances and potential insensitivity of the test equipment, such as the turntable. Overall, the experimental results validate that the designed antenna successfully achieves the four-beam radiation functionality.

## 5. Results

High-gain, low-profile, and low-cost multi-beam scanning antennas with wide-angle beam steering are essential components in next-generation wireless communication systems. This paper presents a metasurface antenna design that employs a microstrip patch antenna with four feed ports as the excitation source. A square metasurface is positioned 0.21λ_0_ above the feed antenna. By sequentially exciting the four ports, the antenna achieves four independently steered beams with good port isolation. Operating at 12.6 GHz, the antenna provides a gain of 10.4 dB for each of the four beams, with an elevation steering angle of 52°. A prototype was fabricated and tested to validate the design. The measured results agree well with the simulations. The antenna not only achieves high-gain beam steering but also features low fabrication cost and a simple architecture. The proposed design offers advantages of structural simplicity, low profile, and cost-effectiveness. This approach, utilizing a square metasurface, combines the benefits of low profile and low cost with flexible control of electromagnetic wave radiation, thereby providing a novel methodology for designing multi-beam communication antennas.

## Figures and Tables

**Table 1:** Geometrical Parameters of the Metasurface Antenna.

Parameter	h_ms	L	W	r	h1
Dimensional (mm)	1.5	55.4	55.4	1.7	1.5
Parameter	W_L	W_d	W1	W_ms	L_ms
Dimensional (mm)	6.2	2	39	8	6.2

## Data Availability

The data supporting this study’s findings are available from the corresponding author upon reasonable request.
